# Shaping the leaf microbiota: plant–microbe–microbe interactions

**DOI:** 10.1093/jxb/eraa417

**Published:** 2020-09-10

**Authors:** Vasvi Chaudhry, Paul Runge, Priyamedha Sengupta, Gunther Doehlemann, Jane E Parker, Eric Kemen

**Affiliations:** 1 Department of Microbial Interactions, IMIT/ZMBP, University of Tübingen, Tübingen, Germany; 2 Max Planck Institute for Plant Breeding Research, Köln, Germany; 3 Institute for Plant Sciences and Cluster of Excellence on Plant Sciences (CEPLAS), University of Cologne, Center for Molecular Biosciences, Cologne, Germany; 4 University of Cologne, Germany

**Keywords:** Biofilm, innate immunity, microbe–microbe interaction, microbial colonization, phyllosphere, quorum sensing

## Abstract

The aerial portion of a plant, namely the leaf, is inhabited by pathogenic and non-pathogenic microbes. The leaf’s physical and chemical properties, combined with fluctuating and often challenging environmental factors, create surfaces that require a high degree of adaptation for microbial colonization. As a consequence, specific interactive processes have evolved to establish a plant leaf niche. Little is known about the impact of the host immune system on phyllosphere colonization by non-pathogenic microbes. These organisms can trigger plant basal defenses and benefit the host by priming for enhanced resistance to pathogens. In most disease resistance responses, microbial signals are recognized by extra- or intracellular receptors. The interactions tend to be species specific and it is unclear how they shape leaf microbial communities. In natural habitats, microbe–microbe interactions are also important for shaping leaf communities. To protect resources, plant colonizers have developed direct antagonistic or host manipulation strategies to fight competitors. Phyllosphere-colonizing microbes respond to abiotic and biotic fluctuations and are therefore an important resource for adaptive and protective traits. Understanding the complex regulatory host–microbe–microbe networks is needed to transfer current knowledge to biotechnological applications such as plant-protective probiotics.

## Teaser

Microbial colonization of above-ground parts of plants is a dynamic and interactive process that requires a high degree of adaptation. Understanding complex host–microbe–microbe interactions is key to new strategies for plant protection.

## Introduction

This review examines how aerial parts of plants, particularly leaves, are colonized by microbes. The first section (‘Colonizing leaf surfaces’) dissects biotic and abiotic factors that shape leaf microbial communities and determine the quality and quantity of colonization. We discuss pre-formed barriers, such as the cuticle, that restrict plant host colonization, and environmental conditions that enhance selection pressures. As a consequence of the extreme conditions on leaves, the properties and generation of biofilms through quorum sensing (QS) are considered. Leaf colonization by microbes is not only impacted by host and environmental factors but also by resident microbes, including pathogens that can severely perturb microbial communities. The second section (‘Microbe–microbe–host interactions’) discusses effects of microbial communities on host susceptibility to pathogens and the impact of plant pathogens and endophytes on microbial host colonization and community composition. A particular focus is on microbe–microbe interactions that are often mediated via the plant host. Individual plant cells also have the capacity to steer microbial activities by pre-formed or induced structures and compounds that influence microbial growth on the leaf surface. The third section (‘Role of the plant immune system in shaping the phyllosphere microbiome’) considers the role of the plant immune system on microbial host interactions with a focus on plant leaf colonization, leaf–microbe outputs, and microbial diversity.

## Colonizing leaf surfaces

### The leaf environment

All terrestrial plants are inhabited by diverse, complex, and interactive communities of microorganisms. With this intimate association, the host plant and its associated microbiota are regarded as a close knit entity and are collectively defined as the holobiont. The holobiont concept implies that evolutionary selection takes place between the host and its associated microbes, and within microbe–microbe members (Vandenkoornhuyse *et al.*, 2015; Hassani *et al.*, 2018; Teixeira *et al.*, 2019). The phenotype of a plant host is the collective outcome of numerous interactions with its microbiota in a particular environment at a time (Vorholt *et al.*, 2017). The ‘phyllosphere’ is referred to as the above-ground portion of plants, dominated by leaves. Its surface represents one of the most abundant habitats on earth (Lindow and Brandl, 2003). Leaves create a fluctuating and unstable environment exposed to multiple stresses and relatively devoid of nutrient sources (Bringel and Couée, 2015). The study of microbial communities inhabiting this stressful leaf habitat and their collective contribution to plant growth, development, and protection has gained intense interest over the last decade. The leaf harbors diverse microorganisms that inhabit the surface and the interior, and are known as epiphytes and endophytes, respectively (Beattie and Lindow, 1999; Lindow and Brandl, 2003). These microorganisms include bacteria as the most common inhabitants, followed by filamentous fungi and yeast strains (Stone *et al.*, 2018), protists (Sapp *et al.*, 2018), and bacteriophages (Balogh *et al.*, 2018). The bacterial titer accounts for ~10^6^–10^7^ cells cm^–2^ of leaf area (Lindow and Brandl, 2003), whereas a typical yeast titer ranges from 10 to 10^4^ cells cm^–2^ of leaf (Shivas and Brown, 1984). The origin of leaf microbial communities is not restricted to a single source. Microbes can colonize the plant leaf vertically through seeds or pollen and horizontally from the air, soil, and insects (Vorholt, 2012; Bodenhausen *et al.*, 2013; Maignien *et al.*, 2014; Bai *et al.*, 2015; Frank *et al.*, 2017).

A stressed and nutrient-poor condition of the leaf surface makes this environment selective to certain microorganisms. Hence, different microbial mechanisms such as ability to extract nutrients, produce hormones and surfactants, as well as motility and biofilm formation can be key to colonization success (Nadakuduti *et al.*, 2012; Ueda *et al.*, 2018; Leveau, 2019; Oso *et al.*, 2019; Streletskii *et al.*, 2019). Most epiphytes survive on the leaf surface by forming large aggregates which help them to cope with the surrounding milieu and maintain a hydrated surface by production of extracellular polymeric substances (EPSs) (Morris and Kinkel, 2002; Lindow and Brandl, 2003; Baldotto and Olivares, 2008; Vorholt, 2012). Other microbes, which are not considered as part of common leaf microbiota, are human commensal or pathogenic bacteria. These can survive and proliferate on the plant leaf, as documented by numerous outbreak studies of human infections on leafy vegetables (Beuchat, 2002; Lindow and Brandl, 2003; Naimi *et al.*, 2003; Islam *et al.*, 2004; Melotto *et al.*, 2006; Munther *et al.*, 2020). Such microbes are able to colonize and survive in an unfavorable leaf environment if they are teamed up with aggregates of pre-colonized leaf microbiota (Monier and Lindow, 2005).

Host-adapted microbial colonizers are more tolerant to abiotic stresses such as harmful UV radiation (Kamo *et al.*, 2018), oxidative stress, and desiccation (Vorholt, 2012), and can utilize nutrients (Crombie *et al.*, 2018) and vitamins (Yoshida *et al.*, 2019) available on the leaf surface. By mitigating biotic and/or abiotic stress(es) and influencing plant growth and fitness, microbes develop adaptive traits and intimate associations with leaves (Vorholt, 2012; Helfrich *et al.*, 2018). Plant host–microbiota interactions are built on the transfer of molecular and genetic information. Important colonization factors, such as secondary metabolites, QS systems, biofilm formation, and cell signaling, are responsible for this exchange of information (Braga *et al.*, 2016; Leveau, 2019; Flores-Núñez *et al.*, 2020). Leaf microbial communities influence plant fitness by modulating the host plant immune system and promoting plant growth in above-ground tissues (Stone *et al.*, 2018).

### Leaf surface structure and chemistry relevant to microbiota assembly

Leaf structure and its surface chemistry create a peculiar microenvironment. During evolution, the formation of a leaf cuticle layer was a prerequisite for land plants to survive out of water. The composition and function of the cuticle is summarized in a review (Müller and Riederer, 2005). The cuticle also covers preferential sites for microbiota colonization, such as the surface of leaf epidermal cells, stomata, and trichomes (Teplitski *et al.*, 2011; Peredo and Simmons, 2018). The cuticle layer is composed of structurally and chemically heterogeneous compounds primarily made of biopolyester cutin, wax, and more minor compounds such as phenolics, cutan, and polysaccharides (Gniwotta *et al.*, 2005; Nawrath *et al.*, 2013). Under constant exposure to abiotic and biotic factors, the epidermal layer of leaf tissues performs its primary function as a protective barrier by preventing seepage of water from the leaf surface as well as external water and solutes from entering the plant. Moreover, the cuticle plays a critical role in mediating interactions with leaf microbiota, including commensal, beneficial, and pathogenic microorganisms (Schönherr, 2006; Vorholt, 2012; Vacher *et al.*, 2016).

Leaf microbiota utilize a number of strategies to enter and penetrate the leaf cuticle. A major route is through natural stomatal openings and wounds resulting from lytic enzymes and osmotic pressure (Frank *et al.*, 2017). Stomata are enclosed by two guard cells to regulate gas exchange and transpiration from the leaf epidermis. Movement of microbes between the external and internal parts of the phyllosphere via stomata has been generally regarded as a passive process, in which the microorganism and plant leaf do not engage in active dialog to permit and/or restrict microbe entry (Underwood *et al.*, 2007). Studies have demonstrated the role of signal transduction cascades in bacterial regulation of stomatal aperture (Zeng *et al.*, 2010; Zheng *et al.*, 2012).

Stomatal aperture is regulated by biotic and abiotic environmental conditions. In general, successful microbial colonization of the leaf depends on stomatal aperture (Ou *et al.*, 2014). Decades of research have shown that phytopathogenic bacteria and fungi exploit stomata as a point of entry for invasion. To breach surface barriers via stomata, host-adapted bacteria subvert plant abiotic stress signaling to suppress stomatal closure during infection (Melotto *et al.*, 2006; Okamoto *et al.*, 2009; Zeng *et al.*, 2010; Xin *et al.*, 2018). To counter pathogen invasion, stomatal guard cells recognize diverse pathogen-/microbe-associated molecular patterns (PAMPs/MAMPs) such as flagellin, chitin, and chitosan (Arnaud and Hwang, 2015). These recognitions and downstream signaling processes lead to closing of stomatal pores and hence prevent bacterial entry as part of the plant immune response. Suppressing the stomatal defense system is an important adaptation mechanism for switching from an epiphytic to an endophytic lifestyle, leading to bacterial disease (Melotto *et al.*, 2017).

Structural and chemical heterogeneity of the leaf cuticle is detected within and between plant genotypes, organs, and even developmental stages (Müller and Riederer, 2005). A role for leaf surface microbiota, together with leaf cuticle mechanisms, was observed in *Arabidopsis thaliana* in resistance against *Botrytis cinerea*, a broad host-range necrotrophic fungal pathogen (Ritpitakphong *et al.*, 2016). Analyses reveal important effects of variation in cuticle chemical and physical composition modulating associations between plants and microbiota, including beneficial and pathogenic microorganisms (Aragón *et al.*, 2017).

Apoplastic spaces inside leaves are large intercellular spaces which mediate gas exchange between cells and are essential for most plant species to achieve efficient photosynthesis (Chen *et al.*, 2020). Humidity controls occupancy of pathogens in apoplastic spaces and is an important initial determinant of leaf colonization (Xin et al., 2016, 2018). Indeed, water availability in the leaf apoplast is a key factor determining successful colonization by neutral and beneficial, but also pathogenic microbes that compete with the host for water (Aung *et al.*, 2018; Chen *et al.*, 2020). It is therefore not surprising that the leaf apoplast has emerged as a decisive environment for host–microbe communication during colonization and for the mobilization of active defense mechanisms to counter pathogen infection.

### Diversity of leaf-colonizing microbiota

Distinct microbiota interactions are found in the leaf compartment which influence the plant host, shaping the microbial community and colonization success. The microbiota in the leaf is not composed of a single species but rather intraspecies, interspecies, and cross-kingdom microbial assemblies of bacteria, yeast, fungi, and protists, establishing the leaf environment (Hardoim *et al.*, 2015). The establishment and abundance of these leaf microbial communities and their distinct effects on the host plant—whether this is commensal, beneficial, or detrimental—are the outcome of numerous interactive processes. These processes are, in turn, influenced by incoming and outgoing microorganisms to and from the leaf habitat and their rate of multiplication, dispersal, and decline in a particular niche (Vorholt, 2012; Wagner et al., 2014, 2016; Lebeis *et al.*, 2015; Vacher *et al.*, 2016; Remus-Emsermann and Schlechter, 2018; Stone *et al.*, 2018; Laforest-Lapointe and Whitaker, 2019).

To gain a better understanding of plant leaf–microbe interactions and outcomes, it is crucial to identify and characterize the microbial community that has evolved and adapted to the leaf environment. There are now ample studies describing the diversity and community structure of leaf-associated microbes, their characterization by next-generation sequencing, culture-independent and culture-dependent methods based on taxonomic markers, and their roles in host development and protection against stress (Romero *et al.*, 2014; Harsonowati *et al.*, 2017; Wallace *et al.*, 2018; Dong *et al.*, 2019). Notably, the leaf-associated microbial community in plants such as common bean (*Phaseolus vulgaris*), lettuce (*Lactuca sativa*), and neotropical forest and poplar trees consists of four major bacterial phyla, namely *Proteobacteria*, *Firmicutes*, *Bacteroidetes*, and *Actinobacteria* (de Oliveira Costa *et al.*, 2012; Rastogi *et al.*, 2012; Kembel *et al.*, 2014; Durand *et al.*, 2018). Rastogi *et al.* (2012) demonstrated variability in bacterial community profiles on field-grown lettuce leaves with respect to time, space, and environment. In characterizing a tropical tree microbiome, Kembel *et al.* (2014) showed that leaf bacterial communities are dominated by *Actinobacteria*, *Alpha*-, *Beta-*, and *Gammaproteobacteria*, and *Sphingobacteria.* The Kembel study also identified microbial correlations with host growth, mortality, and function. In *A. thaliana*, there is a taxonomic and functional overlap between bacterial communities in the leaves and roots, and evidence that soil is the main driver of leaf bacterial community structure (Bai *et al.*, 2015). Phyla belonging to *Proteobacteria*, *Actinobacteria*, and *Bacteroidetes* were found to be most abundant in *A. thaliana*, common ash (*Fraxinus excelsior*), and other tree leaves (Redford *et al.*, 2010; Bodenhausen *et al.*, 2013; Bai *et al.*, 2015; Griffiths *et al.*, 2020; Ulrich *et al.*, 2020).

Redford *et al.* (2010) in a study of leaves of 56 tree species also concluded that interspecies variation is more prevalent than intraspecies variation and that there is a correlation between tree phylogeny and bacterial community composition. Using 16S rRNA, ammonia oxidation (*amoA*), and nitrogen fixation (*nifH*) gene markers, Bao *et al.* (2020) characterized phyllosphere bacteria and established differences in the diversity and composition of bacteria, including diazotrophic communities, over two seasons in three different tree species. An abundance of season-specific bacterial genera highlighted that there might be particular mechanisms of leaf adaptation in different seasons (Bao *et al.*, 2020). However, *Methylobacterium* and *Sphingomonas* species were highly abundant in the plant leaf environment of three species, namely *A. thaliana*, *Trifolium repens*, and *Glycine max* (Delmotte *et al.*, 2009). Furthermore, Durand *et al.* (2018) reported bacterial community members belonging to *Methylobacterium*, *Kineococcus*, *Sphingomonas*, and *Hymenobacter* on the leaf surface of poplar trees. Apart from these, members of the genus *Pseudomonas* are also predominantly found in the phyllosphere of a wide range of plant species (Rastogi *et al.*, 2013).

Numerous studies revealed the association of diverse leaf epiphytic and endophytic filamentous fungi and yeasts with plant host (Arnold *et al.*, 2007; Kharwar *et al.*, 2010; Porras-Alfaro and Bayman, 2011; Sun *et al.*, 2014; Wang *et al.*, 2016; Qian *et al.*, 2018; Yao *et al.*, 2019; Into *et al.*, 2020). Huge diversity, spatial structure, and host association were observed among leaf endophytes and a role in protecting the plant against the devastating foliar oomycete pathogen, *Phytophthora* sp. (Arnold *et al.*, 2003). Qian *et al.* (2018) found that *Dothideomycetes* and *Eurotiomycetes* are dominant members in *Mussaenda pubescens* and identified intraspecific host genetics as primary drivers in shaping regional phyllosphere fungal communities. Yao *et al.* (2019) reported that *Dothideomycetes* and *Tremellomycetes* are dominant members in the mangrove ecosystem in six mangrove species, namely *Aegiceras corniculatum*, *Avicennia marina*, *Bruguiera gymnorrhiza*, *Kandelia candel*, *Rhizophora stylosa*, and *Excoecaria agallocha*, and obtain ecosystem insights for species co-existence and community stability. Using a culture-independent approach, Agler *et al.* (2016) extracted yeast genera belonging to *Protomyces*, *Dioszegia*, *Leucosporidium*, and *Rhodotorula* in the phyllosphere of wild *A. thaliana* populations from Germany. In another study, Dhayanithy *et al.* (2019) characterized fungal endophytes from the leaves and stems of *Catharanthus roseus* and reported *Colletotrichum*, *Alternaria*, and *Chaetomium* genera as common members. *Cladosporium* and *Alternaria* filamentous fungi, *Cryptococcus* and *Sporobolomyces* yeasts, and *Pseudomonas* spp. and *Erwinia herbicola* bacteria were commonly found colonizing leaves of *Beta vulgaris* (Thompson *et al.*, 1993). In another study, by Glushakova and Chernov (2004), changes in epiphytic yeast populations were observed over the year in evergreen common wood sorrel *Oxalis acetosella* L., revealing that species diversity was high in autumn and low in spring. In contrast, *Rhodotorula glutinis* and *Sporobolomyces roseus* species were abundant throughout the year. Interestingly, leaves also harbor certain suppressive bacteria that can restrict phyllosphere bacterial diversity and increase resistance, for example in maize, to Southern leaf blight (SLB) fungal infection (Balint-Kurti *et al.*, 2010). Numerous studies showed the diversity and abundance of yeast in the leaf environment; however, there is a need for more in-depth understanding of the biological mechanisms that showed their role towards host growth and protection.

Sometimes, serious human pathogens such as *Salmonella enterica* serovar *Typhimurium* 14028s (*S. typhimurium* 14028s) and *Escherichia coli* O157:H7 (EcO157) colonize fresh leafy vegetables such as lettuce (*Lactuca sativa*) via damaged leaf tissue and can cause food-borne disease outbreaks (Saldaña *et al.*, 2011; Roy *et al.*, 2013). At the site of injury, lettuce leaf tissue provides substrates for proliferation, and choline which helps the pathogen combat osmotic stress (Scott *et al.*, 2017). It is not clearly understood how human pathogenic/commensal bacteria survive in the extreme environmental conditions encountered by plants. Nevertheless, human pathogens can stay as persister cells also known as cells in a transient dormant state on the plant and cause disease once they encounter a new environment (Munther *et al.*, 2020). In a recent study, Jacob and Melotto (2020) discovered genetic diversity among lettuce genotypes and resident human pathogenic *S. Typhimurium* 14028s and *E. coli* O157:H7, and found a link between genetic diversity and differences in plant immune responses to these bacteria. However, in comparison with human pathogens, less is studied on persister cells in phytopathogen associations with the leaf (Martins *et al.*, 2018). Mechanisms preventing invasion by plant pathogenic microorganisms and plant-induced defense responses are discussed below.

### Role of metabolites in leaf preferential colonization by microbiota

Although present as epiphytes on plant hosts, not all microbes are able to colonize and establish themselves inside leaves. Initial colonization and entry of microbes as a community into a leaf is not a random process in which arbitrary communities adhere and grow, but an organized series of events. Steps involve attachment, movement, and cellular interactions. These steps are facilitated by the leaf surface structure (see above) which regulates colonization as an important priming event in microbial community interactions with the plant (Lebeis *et al.*, 2015; Flemming and Wuertz, 2019). Research hypothesizes that a small community of established microbes associated with the host are inherited vertically through the seed (Nelson, 2018). These microbes are thought to influence recruitment, structuring, and stabilizing of microbiota throughout the plant life cycle (Newcombe *et al.*, 2018).

While several studies have uncovered a role for microbiota at the site of leaf colonization, the functional relationship between leaves and their associated microbial community is poorly understood. Some studies showed production of biosurfactants by epiphytic bacteria on the leaf and the role of these molecules in movement and nutrient acquisition aiding leaf surface adaptation (Bunster *et al.*, 1989; Neu *et al.*, 1990; Hutchison and Johnstone, 1993; Schreiber *et al.*, 2005; Burch et al., 2011, 2012, 2014). Analysis of gain- and loss-of-biosurfactant (Syringafactin) *Pseudomonas syringae* pv. *syringae* B728a strains on bean (*Phaseolus vulgaris*) leaves indicated that this hygroscopic biosurfactant increases diffusion of water across a waxy leaf cuticle surface which attracts moisture and nutrients to benefit the bacteria (Burch *et al.*, 2014). As the initial microbe–leaf contact point, a role for cuticle wax biosynthesis genes in phyllosphere bacterial community composition was observed in *A. thaliana* (Reisberg *et al.*, 2013).

As an abundant genus of leaf microbiota, culturable isolates assigned to *Sphingomonas* sp. were found to provide protection in *A. thaliana* against the foliar pathogen *P. syringae* pv. *tomato* strain DC3000 (Innerebner *et al.*, 2011). Combinatorial metagenome and metaproteome studies conducted on the leaf microbiota of three plant species, namely *A. thaliana*, *T. repens*, and *G. max*, offer clues to leaf microbiota functions and suggest an important role for one-carbon metabolism and transport processes in the microbiota (Delmotte *et al.*, 2009). Methanol is a common one-carbon substrate available to leaf microbiota as a result of the diurnal metabolic cycle and is a pectin methylesterase by-product processed by plants in large amounts during cell wall degradation for growth and development (Fall and Benson, 1996). Methanol-utilizing microorganisms assigned mostly to the genus *Methylobacterium* consume methanol during leaf colonization of numerous plant species, which enhances fitness (Sy *et al.*, 2005; Delmotte *et al.*, 2009; Knief *et al.*, 2010; Sanjenbam *et al.*, 2020). Together with phyllosphere-specific metabolites, leaf-colonizing microbiota offer a unique pool of bioactive metabolites and traits to counter stresses such as UV rays, reactive oxygen species (ROS), and dehydration (Delmotte *et al.*, 2009; Vorholt, 2012; Helfrich *et al.*, 2018). Such traits might become useful in developing probiotics for agriculture.

### Biofilm formation by leaf microbiota

Microbes colonize leaves as complex multicellular communities. Long-term co-evolution of communities that have co-adapted and specialized results in distinct associations which further facilitate mutualistic, symbiotic, competitive, antagonistic, and indeed pathogenic microbial lifestyles with the host (Braga *et al.*, 2016). Association between communities starts with initial adhesion to the leaf surface and ends with a complex network of interactions. Most research on leaf microbiota has focused on bacterial communities which assemble in aggregates of up to 10^4^ cells (Monier and Lindow, 2004). These bacterial clusters are the result of aggregation between multiple cell types or clonal reproduction of a single cell (Tecon and Leveau, 2012). Bacterial surfaces play a critical role in aggregation, biofilm formation, adherence, and survival on leaf surfaces. As a part of a survival strategy, human pathogens also formed aggregates with other bacteria on the leaf, probably affording some protection during their limited survival span (Brandl and Mandrell, 2002).

Biofilms are aggregates of microbial communities in which cells adhere to each other and to a surface enveloped in a matrix of extracellular polymeric compounds, which protects the community under adverse conditions (Davey and O’toole, 2000). In nature, ~70% of bacteria on leaves are found in aggregates which confer a survival and colonization selective advantage over solitary cells on leaf surfaces (Morris and Kinkel, 2002; Monier and Lindow, 2003). Bacterial cell aggregates need to reach a minimum size to gain protection in unfavorable environments. For instance, Monier and Lindow (2003) showed that aggregates of ~100 or more cells are essential for protection against desiccation on plant leaf surfaces. A large pool of microbial communities on the leaf is protected in stress-tolerant aggregates, and dispersal of single cells leads to new microcolonies (Danhorn and Fuqua, 2007). From the attachment of cells to generation of mature biofilms, specific traits such as motility and adhesion are necessary to move and disperse on the leaf, for optimal resilience to biotic and abiotic stresses (Grinberg *et al.*, 2019).

Using atomic force microscopy (AFM), Mittelviefhaus *et al.* (2019) quantified high magnitude differences in adhesion forces of leaf bacteria. Biofilm formation can also be observed in symbiotic and pathogenic lifestyles on plants, and linked with the disease cycle of phytopathogenic bacteria (Bogino *et al.*, 2013). A role for the biofilm in colonization, disease development, and biocontrol activity was established in different studies for *Xanthomonas axonopodis* pv. *citri*, *Xanthomonas vesicatoria*, and *Bacillus amyloliquefaciens* (Malamud *et al.*, 2011; Felipe *et al.*, 2018; Salvatierra-Martinez *et al.*, 2018). Nevertheless, the precise mechanism(s) by which plants regulate biofilm-associated communities are unclear (Ference *et al.*, 2018; Kyrkou *et al.*, 2018). The phytopathogen *X. axonopodis* pv. *citri* forms biofilms on leaves of citrus species during the development of citrus canker disease (Brunings and Gabriel, 2003; Rigano *et al.*, 2007; Malamud *et al.*, 2011). Notably, microbial biofilms are also important for pathogenesis by *Xylella fastidiosa*, the causal agent of devastating Pierce’s disease of grapes, olives, and citrus fruits, which culminates in blockage of the host vascular system (Hopkins, 1989; Marques *et al.*, 2002; Thorne *et al.*, 2006; Rudrappa *et al.*, 2008; Kyrkou *et al.*, 2018). Bacterial brown spot disease of bean leaves caused by *P. syringae* pv. *syringae* was also found to require biofilm formation (Monier and Lindow, 2004). Similarly, motility in biofilm formation was essential for host colonization by phytopathogens such as *Ralstonia solanacearum*, *Pantoea stewartii*, and *Dickeya dadantii* (Tans-Kersten *et al.*, 2001; Herrera *et al.*, 2008; Jahn *et al.*, 2008). Much biofilm research has concentrated on specific groups of microorganisms with emphasis on bacteria. These studies emphasize the importance of biofilms in the survival and colonization of leaves by both damaging and potentially beneficial microbe communities under unfavorable conditions.

### Quorum sensing in leaf microbiota

Microbial colonization to plants is regulated by the density-dependent QS phenomenon, a strategy to survive in the challenging leaf habitat. QS mechanisms involve intra- and also interspecies bacterial communication to share information and regulate their physiological activities and coordinate gene expression of factors such as motility, biofilm, host colonization, and virulence (Ng and Bassler, 2009; Elias and Banin, 2012). Different bacterial groups synthesize and use particular chemical signals or QS molecules for communication. For example, Gram-negative bacteria employ *N*-acyl-l-homoserine lactone [AHL; also called autoinducer-1 (AI-1)] and quinolones as QS molecules, whereas modified oligopeptides (autoinducer peptides, AIPs) are commonly used by Gram-positive bacteria for communication between cells (Taga and Bassler, 2003). Other autoinducers belonging to boron furan-derived QS molecules or AI-2 are specifically for interspecies communication (Federle, 2009). Diffusible signal factor (DSF) is another family of conserved QS signals utilized for the regulation of virulence factor in numerous Gram-negative bacterial pathogens (Li *et al.*, 2019).

Interestingly, these QS molecules can also be recognized by cells of eukaryotes, including plants and fungi (Dudler and Eberl, 2006; Irie and Parsek, 2008). QS signal information during initial leaf surface colonization is highly localized and the quorum area can be as low as 10 cells (Gantner *et al.*, 2006; Dulla and Lindow, 2008). AHL QS signaling molecules occur naturally in the leaf environment and might impact leaf–bacteria interactions (Enya *et al.*, 2007). Lv *et al.* (2012) screened a number of AHLs produced by Gram-negative *Proteobacteria* as QS signals in the tobacco phyllosphere and monitored bacterial community composition. Here, *Pseudomonas* and other AHL-producing *Gammaproteobacteria* were found to use QS signals for survival and protection against other epiphytic members in the nutrient-limited phyllosphere environment. It is therefore likely that AHL QS signaling can also limit pathogenic microbes on leaves. On the other hand, in some phytopathogens, intraspecies QS was studied; for instance in *Xanthomonas* associated with grapevines, QS molecules control the expression of virulence factor as well as biofilm formation (Danhorn and Fuqua, 2007). For *Pseudomonas syringae* in tobacco and bean interaction, QS mediated control of motility and exopolysaccharide synthesis was observed for their role in biofilm formation and colonization of bacteria on leaf (Quiñones *et al.*, 2005).

In this section, we covered microbial community composition and diversity, and colonization, survival, and adaptation in a leaf habitat. There are still significant gaps in knowledge of the types of microbial interaction, and mechanisms of competition and cooperation between leaf microbiota members, that facilitate microbial community stability and structure.

## Microbe–microbe–host interactions

### Communication between pathogenic microbes

Infection by pathogens can have a significant impact on the resident leaf microbial community. For example, severe SLB disease was correlated with reduced species richness in the epiphytic bacterial population of maize (Manching *et al.*, 2014). Pathogenic microbes can also increase the susceptibility of their host plant to colonization by other microbes, which would not normally be invasive. For example, *Albugo candida* (white rust) enhanced susceptibility of various *Brassicaceae* species to fungal mildew pathogens (Cooper et al., 2002, 2008). In turn, white rust disease symptoms caused by *A. candida* in *Brassica juncea* were elevated by subsequent inoculation with the downy mildew pathogen *Hyaloperenospora parasitica*, which normally colonizes *B. juncea* asymptomatically and thereby increases its susceptibility towards white rust (Kaur *et al.*, 2011). A similar mutual infectivity relationship was found in *A. thaliana*, in which an adapted oomycete pathogen, *Albugo laibachii*, induced susceptibility to the non-host pathogen *Phytophthora infestans* (Belhaj *et al.*, 2017). Therefore, *Albugo* infections in *Brassicaceae* in some way promote a host jump by certain pathogens (Thines, 2014).

While several reports highlight the importance of interspecies microbial communication in disease development, it should be emphasized that different cells of a single pathogenic microorganism behave distinctly to orchestrate successful colonization of the host. This was shown for hyphal cells of the fungal pathogen *Sclerotinia sclerotiorum*, which have differential gene expression patterns and metabolic heterogeneity during successful colonization of host plants (Peyraud *et al.*, 2019).

QS between pathogenic microbes leads to an increase in virulence and pathogenicity in the host plant. QS-based systems of the *Pseudomonas savastanoi* pv. *savastanoi* (olive knot pathogen) and *Erwinia toletana* (olive knot cooperator) stabilize the community and exchange QS signals, and this cooperation results in a more aggressive disease on olive plants (*Olea europaea*) (Caballo-Ponce *et al.*, 2018). An intriguing example of QS mediating pathogen infection in eukaryotes was reported for the oomycete pathogen *Phytophthora nicotinae.* Zoospore-derived extracellular fluids contain QS components which induced zoospore aggregation that increased pathogen infectivity (Kong *et al.*, 2010).

### Interactions of foliar pathogens with endophytes

Endophytes are microorganisms which colonize the internal organs of the plant without causing visible symptoms. In several cases, endophytic microbes were reported to impact plant stress protection and development. Plant pathogens can be inhibited by a number of mechanisms, for example hyperparasitism, competition, and/or antibiosis (Busby *et al.*, 2016). Fungal endophytes promoted induction of phenolic compounds in perennial ryegrass, thereby providing resistance against pathogenic growth (Pańka *et al.*, 2013). Direct interactions are also observed between endophytes and pathogens. Fungal endophytes of oak tree were found to be possible antagonists of *Erysiphe alphitoides*, the causal agent of powdery mildew disease (Jakuschkin *et al.*, 2016). *Metarhizium robertsii* colonizes insect larvae present in the plant root tissue and transfers nutrients from the insect to the host (Branine *et al.*, 2019). Biosynthetic gene clusters including non-ribosomal peptide synthetase (NRPS) and polyketide synthase (PKS) genes were identified in endophytes (Miller *et al.*, 2012; Ludlow *et al.*, 2019), which might contribute to their biocontrol potential.

Busby *et al.* (2015) reported that disease modification is an ecological function shared by common foliar fungi of *Populus trichocarpa*. Species of *Cladosporium* and *Trichoderma* were identified to be antagonists of *Melampsora* rust pathogen in wild *P. tricocarpa* populations. These results differ from previous studies by Raghavendra and Newcombe (2013) where *Stachybotrys* sp., *Trichoderma atroviride*, *Ulocladium atrum*, and *Truncatella angustata* have been reported to induce quantitative disease resistance in *P. trichocarpa* against *Melampsora* rust pathogen under controlled experimental conditions. On the contrary, the above fungi were found to be quite rare in wild *P. trichocarpa* (Busby *et al.*, 2015) and this hints at the disparity between disease-modifying action of foliar fungi under wild and experimental conditions.

Endophytes utilize QS to act against pathogenic microbes by expressing QS inhibitors (QSIs) to attenuate the activity of AIs, or quorum quenching (QQ) enzymes to disrupt signaling molecules. For example, AHL lactonase enzyme (a potent quorum quencher) present in endophytic bacteria has been reported to inhibit the plant pathogens *Erwinia carotovora* (Dong et al., 2000, 2001), *Bacillus* sp., subspecies of *Bacillus thuringiensis* (Lee *et al.*, 2002; Ulrich, 2004), and *Enterobacter asburiae* (Rajesh and Ravishankar Rai, 2014). Ma *et al.* (2013) explored the diversity of tobacco (*Nicotiana tabacum*) leaf-associated strains with QQ activity for disruption of AHL-mediated QS, by using the biosensor reference strain *Chromobacterium violaceum* CV026. These bacterial quorum quenchers can be used as effective biocontrol agents against plant pathogens (Ma *et al.*, 2013). More research is needed to understand how these interactive chemical processes impact plant microbiota community structure and function on plant hosts, and their consequences for plant health.

### Microbial succession in host interactions

There is a constant struggle between different microorganisms residing inside the plant for nutrients, space, and survival. In this arena, the order of arrival of microbes can be a decisive factor between host disease resistance and facilitation. *In planta* experiments on *Phaseolus lunatus* have shown that, if a pathogen was introduced to the plant on the same day or before inoculation with an endophyte, disease resistance was more strongly reduced than when the endophyte had already colonized the host (Adame-Alvarez *et al*., 2014).

The situation is reversed in the case of the biotrophic maize smut fungus *Ustilago maydis* which is inhibited by the endophyte *Fusarium verticillioides* when both organisms are co-inoculated to the plant. Pre-inoculation with the endophyte had no impact on disease severity, whereas post-inoculation caused greater disease progression and decreased plant growth (Lee *et al.*, 2009). This result suggests that *F. verticillioides* can inhibit *U. maydis* by direct interaction and not by induction of host defense responses. In line with this, the presence of *U. maydis* does not result in a significant difference in the diversity of the endophytic community, causing small localized differences in the community structure because of infection (Pan *et al.*, 2008). Also, variation of the endophytic community does not correlate with levels of resistance to *U. maydis* in different maize lines (Pan *et al.*, 2008).

### Microbial lifestyle in host interactions

While so far there is no direct evidence for modulation of *U. maydis* infection with endophytic microbes, the group of smut fungi themselves represents an interesting example of organisms which exhibit different lifestyles in different niches. Generally, basidiomycete yeasts are present abundantly in the leaf microbial community of *A. thaliana*, along with other endophytic bacteria, as well as oomycetes (Agler *et al.*, 2016). The anamorphic yeast *Moesziomyces albugensis* was recently found to antagonize *Albugo laibachii* infection and reduce disease development on plants (Eitzen *et al.*, 2020, Preprint). This happens to differ from previous studies by Agler *et al.* (2016), where the presence of the basidiomycete yeast, *Dioszegia* sp., was positively correlated with the oomycete, *A. laibachii*.

*Moesziomyces* sp. (classified as *Pseudozyma* sp. until phylogenetic reconstruction by Wang *et al.*, 2015) belong to the order of *Ustilaginales*, and have been reported to act as biocontrol agents in a number of cases (Barda *et al*., 2014; Gafni *et al.*, 2015). Comparative transcriptomics identified a secreted hydrolase of *M. albugensis* being induced on the Arabidopsis leaf surface in the presence of *A. laibachii*, and reverse genetics demonstrated that the antagonism of *M. albugensis* towards *A. laibachii* depends on the expression of this enzyme (Eitzen *et al.*, 2020, Preprint).

Such insights into the functional basis of microbial interactions of the *Ustilaginales*, where members of the same species can either be plant pathogens or beneficial epiphytes, show us that there might be no clearly demarcated barrier between organisms which behave as a pathogen and a plant-protecting microbe (i.e. a pathogen’s antagonist). Similarly, different strains of the pathogenic fungus *Fusarium oxysporum* can act as microbial antagonists against other *F. oxysporum* strains (van Dam *et al.*, 2016). The differences in their lifestyle have traced back to the effector repertoire, with the epi-/endophytic strains having fewer or no host-specific effectors (de Lamo and Takken, 2020). *Moesziomyces* sp., however, encodes a fully equipped set of effector genes (Eitzen *et al.*, 2020, Preprint), including a functional homolog of the *U. maydis* core virulence effector Pep1 (Sharma *et al.*, 2019). This evidence suggests that anamorphic *Ustilaginales* yeasts have the potential to form infectious filamentous structures (Kruse *et al.*, 2017) and at the same time raises the question of which factors drive the adaptation of these organisms to either a pathogenic or an epiphytic lifestyle.

Knowledge of the roles of microbe–microbe–host interactions in determining microbial invasiveness will aid understanding of the cross-domain interactions in pathogenicity. Nevertheless, more fundamental research is needed to disentangle microbe–microbe and microbe–host interactions at the level of individual strains to determine what underpins functional microbial assemblies in nature.

## Role of the plant immune system in shaping the leaf microbiome

The plant innate immune system comprises a large repertoire of plasma membrane-localized (surface) and intracellular receptors which recognize microbial or modified host molecular signatures and retain plant health and secure plant propagation. Surface immune receptors (often referred to as pattern recognition receptors, or PRRs) are members of a diverse family of ligand-binding proteins that sense microbial, environmental, developmental, and nutritional cues (Saijo *et al.*, 2018; Cheng *et al.*, 2019). In terms of shaping microbial communities, it is the PRR activities that are thought to gate microbial entry into leaf tissues, and effectively ward off colonization by host non-adapted strains (Boutrot and Zipfel, 2017). The intracellular receptor panels [consisting mostly of nucleotide-binding/leucine-rich repeat (NLR) proteins] are similarly diverse and are selected as triggers of strain-specific resistance to host-adapted pathogens (Eitas and Dangl, 2010; Jones *et al.*, 2016; Wu *et al.*, 2017; Burdett *et al.*, 2019; de Weyer *et al.*, 2019).

**Fig. 1.**
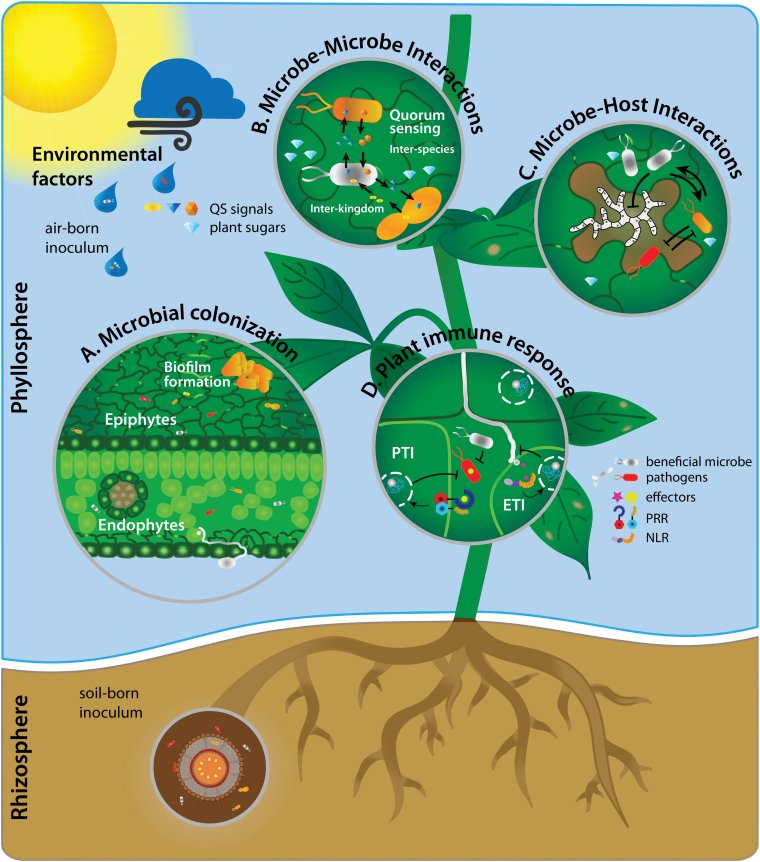
Microbial colonization of the above-ground part of the plant (phyllosphere), as well as the below-ground part (rhizosphere). (A) The microbial colonization on the leaf takes place on the leaf surface (epiphytes) from air-borne and soil-borne inocula and the inner leaf part (endophytes). Microbial colonization can lead to exogenous intraspecies biofilm formation on the leaf surface. (B) Microbe–microbe interactions occur between interspecies and interkingdoms, referred to as quorum sensing. Quorum-sensing molecules impacting microbial recognition and biofilm formation on leaves. (C) Pathogenic microbes colonize host plants by means of their virulence. The genetic make-up of both the host and pathogen contributes to disease progression. However, other microbes in the host phyllosphere can influence this plant–pathogen interaction by either facilitation or antagonism. (D) Plant immune responses are of specific interest as host–microbe interactions shaping the phyllosphere microbiome. Non-host-adapted pathogens are involved in PAMP-triggered immunity (PTI) and recognized via pattern-recognition receptors (PRRs). Host-adapted microbes are recognized via nucleotide-binding leucine-rich repeat receptors (NLRs), summarized in effector-triggered immunity (ETI).

The activation of plant immune responses by mobilizing a network of defense and stress hormone pathways has been extensively characterized in binary plant–pathogen interactions (Noman *et al.*, 2019; Zhao *et al.*, 2019). Little is known about the impact of plant immunity signaling networks on host–microbe interactions in leaf microbial communities (Fig. 1D). High-throughput DNA and RNA sequencing of leaf samples from natural environments have enabled examination of complex microbial communities in plant-specific niches in time and space (Agler *et al.*, 2016). Analysis of microbial metadata and their integration with experimental testing should provide a clearer picture of the role of plant immunity signaling in shaping leaf microbial community structure and, in turn, how resident microbes influence host immunity.

In this section, we consider evidence that abiotic and biotic stress responses modulate microbial consortia on leaves and discuss the consequences for plant fitness. It is becoming clear that microbial community structure throughout a plant host’s life cycle is dynamic and modulated by the innate immune system, which itself is tuned to environmental changes.

### The role of pattern-triggered immunity in shaping the leaf microbiota

Most microorganisms on plant leaves are non-pathogenic. However, a broad range of microbes are able to prime innate plant immunity to counter subsequent pathogen attacks (Ritpitakphong *et al.*, 2016; Vogel *et al.*, 2016). Many microbes are recognized by terrestrial plants through their MAMPs initiating pattern-triggered immunity (PTI) responses. PTI is an induced and often low-level but broadly effective resistance response involving phytohormone signaling, secretion of antimicrobial compounds, generation of ROS and mitogen-activated proein (MAP) kinase cascades, and stomatal closure (Bigeard *et al.*, 2015; Bi and Zhou, 2017). Notably, the phytohormone ethylene is required for ROS production in PTI, for example in Arabidopsis resistance to *P. syringae* bacteria and rice resistance to the rice blast fungus, *Magnapothe oryzae* (Mersmann *et al.*, 2010; Guan *et al.*, 2015; Helliwell *et al.*, 2016; Yang *et al.*, 2017). In Arabidopsis, an *ethylene-insensitive2* (*ein2*) mutant displayed an altered bacterial leaf community compared with wild-type plants, suggesting that ethylene signaling is important for modulating the leaf microbiota (Bodenhausen *et al.*, 2014; Nascimento *et al.*, 2018).

A recent study by Chen *et al.* (2020) provided experimental evidence that PTI signaling controls the diversity of endophytic leaf microbiota in microorganism-rich environments. An Arabidopsis quadruple mutant [*min7 fls2 efr cerk1* (*mfec*)] that is defective in PTI and the MIN7 vesicle trafficking pathway (affecting the aqueous apoplastic microenvironment) and a *constitutively activated cell death1* (*cad1*) mutant had altered endophytic bacterial leaf diversity (Chen *et al.*, 2020). In particular, the relative abundance of the bacterial phyla *Firmicutes* was significantly reduced, whereas *Proteobacteria* became the dominating bacterial community members in the mutant plants. The occurrence of PTI components *MIN7* and *CAD1* across major plant lineages suggests that a number of common pathways might govern endophytic microbial proliferation of certain taxa in leaves.

Further research has revealed the importance of resident *Pseudomonas* sp. (*Proteobacteria*) in protecting Arabidopsis against infection by a fungal necrotrophic pathogen, *B. cinerea* (Ritpitakphong *et al.*, 2016). Notably, prominent bacterial clades from soil microbiota such as filamentous *Actinobacteria* (*Strepotmycetes* sp.) are able to activate plant biosynthesis of salicylic acid (SA) and promote leaf defense responses against fungal pathogens (Vergnes *et al.*, 2020). These findings highlight actions of soil-borne microbial inocula of leaves on immunity (Bakker *et al.*, 2013; Haney *et al.*, 2018; Vannier *et al.*, 2019).

The above studies emphasize the role of both commensal and pathogenic microbes in priming PTI as a barrier to colonization of the leaf compartment by host non- or poorly adapted pathogens. Nevertheless these host–microbe interactions were examined mostly under controlled laboratory conditions. Further research is needed to gain an understanding of how PTI shapes plant immune responses and microbiota communities in nature.

### Leaf effector-triggered immunity as a potential microbial gateway

Strain-specific resistance, known as effector-triggered immunity (ETI), is often mediated by intracellular NLR receptors which recognize certain pathogen-delivered virulence factors (effectors) to induce immunity (Monteiro and Nishimura, 2018; Seong *et al*., 2019; Feehan *et al.*, 2020). Pathogen effector-activated NLRs accelerate and amplify many PTI responses, often resulting in host-localized cell death (a hypersensitive response) and rapid pathogen containment (Peng *et al*., 2018). Expressed NLR genes in roots are observed in dicot plant species such as the legume Lotus (Lai and Eulgem, 2018). This in contrast to tested *Brassicaceae* species including *A. thaliana* and the crop oilseed rape (*Brassica napus*), which favor NLR expression in the phyllosphere (Munch *et al.*, 2018). Although NLR activation and downstream signaling mechanisms are becoming resolved, the extent to which this layer of protection against pathogens shapes plant microbial communities is hardly understood.

Diverse microbial communities in leaves can be controlled directly through pathogen colonization on the host or indirectly by host–microbe interactions involving the innate immunity network (Agler *et al.*, 2016). Thus, pathogenic microbes can act as highly interconnected community members (so-called ‘hub microbes’) that dominate microbial community assemblies. For example, the causal agent of white rust on Arabidopsis, *Albugo* sp., appears to act as a hub which alters epiphytic and endophytic bacterial colonization of leaves (Agler *et al.*, 2016; Ruhe *et al.*, 2016). Perturbations of microbial communities by host-adapted biotrophic pathogens such as *Albugo* and *Hyaloperonospora arabidopsidis* (*Hpa*) reduce microbial diversity within leaf habitats and stabilize microbial communities among wild plants (Karasov *et al.*, 2019, Preprint). Hence, microbial diversity can be used as an indicator for microbial community imbalance (Chen *et al.*, 2020).

Whether ETI reactions directly lead to defense priming is not well studied, although in Arabidopsis one important ETI branch leads to a reinforcement and spread of pathogen resistance (so-called basal immunity) in leaf tissues (Lapin *et al.*, 2020). A recent study by Levy *et al.* (2018) analyzed >3800 genomes of plant-associated (pathogenic and non-pathogenic) bacteria. The analysis identified plant-mimicking protein domains (named PREPARADOS) that carry non-canonical ‘embedded’ NLR domains. An increasing number of NLR-fused domains are related to authentic effector targets. PREPARADOS are highly abundant in the bacterial families *Bacteroides* and *Xanthomonadaceae* (Frank, 2019). These findings point to potential interactions between commensal and or pathogenic bacteria with intracellular receptors in host plants. Additional studies are needed to test this hypothesis and dissect functional relationships between NLR panels and the leaf microbiota.

### Stability of microbial consortia against pathogen perturbation

The plant and its associated microbiota is not a static environment but is altered by numerous factors including host genotype, environmental fluctuations, surrounding macro- and microorganisms, and geographical location and associated local variables such as climate (Laforest-Lapointe *et al.*, 2016; Poudel *et al.*, 2016; Wagner *et al.*, 2016; Singh *et al.*, 2018). The stability of a leaf microbial community is measured as the ability to maintain a stable equilibrium state (homeostasis) under biotic or abiotic perturbations (Thébault and Fontaine, 2010). Generally, higher community complexity in a network reflects a more stable community structure (Mougi and Kondoh, 2012). Stable microbial communities or consortia have greater ability to resist perturbation (Ives *et al.*, 2000; Luo *et al.*, 2019; Morella *et al.*, 2020). Studies using culture-independent DNA sequencing revealed similar microbial community patterns in successive year samplings (Copeland *et al.*, 2015). In the phyllosphere, microbial communities can often undergo drastic changes and establish a distinctive and less diverse community (Manching *et al.*, 2014; Copeland *et al.*, 2015). Different computational and experiment-based approaches have been used to capture microbial community homeostasis or deviations over time. Computational microbial network analysis and mining of core microbes are valuable in understanding the factors underlying microbial resilience to controlled perturbations (Astudillo-García *et al.*, 2017; Lemanceau *et al.*, 2017). Much less is known about the dynamics and stability of leaf microbiomes in the field since there is a lack of high-resolution experimental data linked to plant disease and health with respect to time, space, and environmental scale. In recent studies, leaf diseases were linked to disruption of microbial community network stability, resulting in ecosystem dysfunction (Kerdraon *et al.*, 2019; Luo *et al.*, 2019; Leopold and Busby, 2020, Preprint). Understanding how a microbial community corrects itself under conditions of environmental stress is crucial to harness its potential in probiotic applications against aggressive plant pathogens and to track plant-associated human pathogen outbreaks.

### Does immunity priming affect microbial leaf communities?

Various abiotic and biotic factors impact dynamic changes on microbial leaf communities as depicted in the modes of microbial colonization, microbe–microbe, and microbe–host interactions (see Fig. 1 and Table 1). Nevertheless, fundamental mechanisms of microbial community assembly remain barely understood. One major goal of current microbiome research is to understand how microbial consortia in nature secure plant protection during pathogen perturbation. Immunity priming (IP) effects through abiotic (applied chemical compounds) and biotic (biocontrol agents) stimuli seem to play an important role in managing abiotic stress tolerance and disease resistance (Kumar and Verma, 2018). IP has been described as a ‘positive cost–benefit balance in times of stress’ (Martinez-Medina *et al.*, 2016). IP induction involves the phytohormones SA and jasmonic acid (JA), and pipecolic acid-derived signaling molecules that are known to mediate systemic acquired resistance, as well as the non-protein amino acid defense primer β-aminobutyric acid (BABA) (Martinez-Medina *et al.*, 2016). BABA was found naturally in Arabidopsis experiencing abiotic stress (high salinity) and biotic stress, induces broad-spectrum pathogen resistance (Thevenet *et al.*, 2017; Buswell *et al.*, 2018). Another interesting IP compound, (R)-β-homoserine (RBH), primes ethylene and JA pathways and is effective against necrotrophic pathogens such as *B. cinerea* in tomato and *Plectosphaerella cucumerina* (Buswell *et al.*, 2018). Also, brassinosteroids (BRs) have been discussed as factors in an IP mechanism that balances the trade-off between immunity and growth (Yu *et al.*, 2018). These findings highlight the potential utility of chemical compounds for IP. They also prompt studies of how IP impacts leaf microbial diversity under conditions of abiotic and biotic stress.

**Table 1.** Summary of important studies associated with the leaf microbiome

Host plant	Leaf microbiota/leaf microbe under study	Perturbation	Key findings	Reference
Microbial colonization				
*Arabidopsis thaliana*	Bacteria	–	Phyllosphere community profile of *A. thaliana* wild-type Landsberg erecta (Ler) and *eceriferum* (*cer*) mutants (*cer1*, *cer6*, *cer9*, and *cer16*) involved in cuticle biosynthesis. Plant cuticular wax composition affects the phyllosphere bacterial community.	Reisberg *et al.* (2013)
Faba bean (*Vicia faba* L.) and *Arabidopsis thaliana*	*Pseudomonas syringae* DC3118, a coronatine-deficient mutant of *Pseudomonas syringae DC3000*	*–*	In a specific environmental setting, leaf surface colonization by bacteria correlated with stomatal aperture regulation.	Ou *et al.* (2014)
Bean (*Phaseolus vulgaris* L.)	*P. syringae* pv. syringae B728a	*–*	Biosurfactant, syringafactin, produced by *P. syringae* pv. *syringae* B728a on leaves adsorbed on waxy leaf cuticle surface. Provide benefit to bacteria by attracting moisture and aid in nutrient availability.	Burch *et al.* (2014)
*Arabidopsis thaliana*	*Pseudomonas syringae* DC3000	*–*	Humidity-controlled, pathogen-guided establishment of an aqueous intercellular space (apoplast) as an important step in leaf bacterial infection.	Xin *et al.* (2016)
**Microbial composition and diversity**				
Sugar beet (*Beta vulgaris*)	Bacteria, yeasts, and filamentous fungi	–	Seasonal dynamics over a growing season. Fungi: *Cladosporium* and *Alternaria* sp.	Thompson *et al.* (1993)
			Yeast: *Cryptococcus* and *Sporobolomyces* Bacteria*: Pseudomonas* sp. and *Erwinia herbicola*	
Cacao (*Theobroma cacao*)	Fungi (endophytes)	*Phytophthora* sp.	High diversity, spatial structure, and host affinity among foliar endophytes. Endophyte-mediated protection against foliar pathogen.	Arnold *et al.* (2003)
Common wood sorrel (*Oxalis acetosella* L.)	Yeast (epiphytes)	–	Seasonal dynamics of yeasts. Species diversity—maximum in autumn; minimum in spring.	Glushakova and Chernov (2004)
			*Rhodotorula glutinis* and *Sporobolomyces roseus* species abundant throughout the year.	
Loblolly pine (*Pinus taeda*)	Fungi (endophytes)	–	High diversity of foliar fungal endophytes.	Arnold *et al.* (2007)
*Arabidopsis thaliana*, *Trifolium repens*, and *Glycine max*	Bacteria	–	Metaproteogenomic analysis found consistency in three plant species.	Delmotte *et al.* (2009)
			High abundance of *Sphingomonas* sp. and *Methylobacterium* sp.	
			Important role of the one-carbon metabolism and transport processes in the microbiota.	
Tree species	Bacteria (epiphytes)	–	In trees, interspecies variation is more than intraspecies variation in bacterial communities.	Redford *et al.* (2010)
			Correlation between tree phylogeny and bacterial community composition.	
Maize	Bacteria (epiphytes)	Southern leaf blight (SLB)	A specific set of epiphytic bacteria can restrict phyllosphere bacterial diversity and increase resistance to Southern leaf blight (SLB) fungal infection.	Balint-Kurti *et al.* (2010)
*Eucalyptus citriodora* Hook	Fungi (epiphytes and edophytes)	–	Total 33 fungal species assigned to 33 taxa (endophytes, 20; epiphytes, 22).	Kharwar *et al.* (2010)
			Difference in frequency of colonization. Antagonism against human and plant pathogen.	
Lettuce	Bacteria	–	Bacterial community composition by pyrosequencing. Proteobacteria, Firmicutes, Bacteroidetes, and Actinobacteria—most abundant phyla. Insights on variability in bacterial community profile with respect to time, space, and environment.	Rastogi *et al.* (2012)
Common bean (*Phaseolus vulgaris*)	Bacteria (endophytes)	–	158 culturable endophytic bacteria. Phyla distribution 36.7% Proteobacteria, 32.9% Firmicutes, 29.7% Actinobacteria, and 0.6% Bacteroidetes	de Oliveira Costa *et al.* (2012)
*Arabidopsis thaliana*	Bacteria (epiphytes and endophytes)	–	*Proteobacteria*, *Actinobacteria*, and *Bacteroidetes* were found most abundant. Massilia and Flavobacterium are prevalent genera	Bodenhausen *et al.* (2013)
Tomato (*Solanum lycopersicum* L.)	Bacteria (epiphytes)	–	Members of endophytic bacterial communities of tomato leaves exert multiple effects on growth and health of tomato plants.	Romero *et al.* (2014)
Neotropical forest	Bacteria	–	Dominated bacterial communities: Actinobacteria, Alpha-, Beta-, Gammaproteobacteria, and *Sphingobacteria*. Correlation of bacterial community with host growth, mortality, and function.	Kembel *et al.* (2014)
*Arabidopsis thaliana*	Bacteria	–	Taxonomic and functional overlap of leaf and root bacterial communities. Soil as main driver for bacterial members..	Bai *et al.* (2015)
Rice (*Oryza sativa* L.)	*Actinomycetes*	*Pyricularia oryzae* (syn. *Magnaporthe oryzae*)	Rice phyllosphere-associated actinomycetes produce bioactive compounds and control leaf blast disease caused by *Pyricularia oryzae.*	Harsonowati *et al.* (2017)
Sugar maple (*Acer saccharum*)	Bacteria and fungi (epiphytes and endophytes)	–	Microbial communities at the edge of the species’ elevational range differ from those within the natural range.	Wallace *et al.* (2018)
Poplar tree	Bacteria and fungi (epiphytes and endophytes)	Mercury	*Methylobacterium*, *Kineococcus*, *Sphingomonas*, and *Hymenobacter* on the leaf surface.	Durand *et al.* (2018)
*Mussaenda pubescens* var. *alba*	Fungi	–	*Dothideomycetes* and *Eurotiomycetes* are dominant members. Intraspecific host genetic identity, primary driver in shaping regional phyllosphere fungal communities.	Qian *et al.* (2018)
*Arabidopsis thaliana*	Bacteria	–	Determined biosynthetic potential of 224 bacterial strains from Arabidopsis leaf microbiome. Phyllosphere as a valuable resource for the identification and characterization of antibiotics and natural products.	Helfrich *et al.* (2018)
Tomato (*Solanum lycopersicum* L.)	Bacteria (epiphytes)	–	Comprehensive view of the tomato-associated bacterial community.	Dong *et al.* (2019)
			Isolation of beneficial bacterial for future functional studies.	
Mangrove	Fungi (epiphytes and endophytes)	–	*Dothideomycetes* and *Tremellomycetes* are dominant members. Plant identity significantly affects endophytic but not epiphytic fungi.	Yao *et al.* (2019)
*Catharanthus roseus*	Fungi (Endophytes)	–	*Colletotrichum, Alternaria*, and *Chaetomium* are common genera.	Dhayanithy *et al.* (2019)
**Biofilm**				
Common bean (*Phaseolus vulgaris*)	*P. syringae* pv. *syringae*	–	Cause of brown spot disease of bean leaves was the result of biofilm formation of *P. syringae.*	Monier and Lindow (2004)
*Citrus limon* ‘Eureka’	*Xanthomonas axonopodis* pv. *citri*	*–*	Motility and role of flagellum is required for mature biofilm and canker development.	Malamud *et al.* (2011)
Tomato (*Solanum lycopersicum* L.)	*Xanthomonas vesicatoria*	–	Aggressiveness of Xv strains correlated with their ability to move by flagella or type IV pili, adherence to leaves and form well-developed biofilms, help in improved phyllosphere colonization.	Felipe *et al.* (2018)
Tomato (*Solanum lycopersicum* L.)	*Bacillus amyloliquefaciens*	*Botrytis cinerea*	Reduction of biocontrol of BBC 023 on leaves due to its limited ability to generate robust biofilms and colonization in the phylloplane.	Salvatierra-Martinez *et al.* (2018)
**Quorum sensing**				
Tomato (*Solanum lycopersicum* L.)	Bacteria	–	Culturable leaf-associated bacteria community with BCA activity against tomato disease have the ability to produce AHL and IAA.	Enya *et al.* (2007)
Tobacco (*Nicotiana tobacum*)	Epiphytes	–	AHLs induced variation in the bacterial community composition. *Pseudomonas* and other AHL-producing Gammaproteobacteria use QS signals for their survival and protection.	Lv *et al.* (2012)
Tobacco (*Nicotiana tobacum*), common bean (*Phaseolus vulgaris*)	*Pseudomonas syringae*	–	QS-mediated control of motility and exopolysaccharide synthesis was observed for their role in biofilm formation and colonization of bacteria on leaf.	Quiñones *et al.* (2005)
**Microbe–microbe–host interactions**				
*Arabidopsis thaliana*	*Hyaloperonospora parasitica* subsp., *Arabidopsis thaliana*, *H. parasitica* subsp. *Brassica oleracea*, *Bremia lactucae*, and *Albugo candida*	–	*Albugo candid*a suppressed defense signaling pathways in the host, facilitating sporulation by the incompatible downy mildews	Cooper *et al.*, (2002)
*Quercus robur* L.	Foliar fungi and bacteria	*Erysiphe alphitoides*	Direct interaction between *E. alphitoides* and 13 fungal and bacterial operational taxonomic units (OTUs). Fungal endophytes *Mycosphaerella punctiformis* and *Monochaetia kansensis* could be possible antagonists of *E. alphitoides*.	Jakuschkin *et al.* (2016)
*Arabidopsis thaliana*	*-*	*Phytophthora infestans: Albugo laibachii*	Prior colonization of host by *A. laibachii*, helps *P. infestans* to infect an essentially non-host plant.	Belhaj *et al.*, (2017)
*Phaseolus lunatus*	Endophytic fungi for e.g. *Rhizopus*, *Fusarium*, *Penicillium*, *Cochliobolus*, and *Artomyces* spp.	*Pseudomonas syringae* pv. *syringae*, *Enterobacter* sp. strain FCB1, and the fungus *Colletotrichum lindemuthianum*	Order of arrival of fungal endophytes and pathogens on the plant surface can determine disease resistance or facilitation.	Adame-Alvarez *et al*. (2014)
*Zea mays*	Endophyte *Fusarium verticillioides*	*Ustilago maydis*	*F. verticillioides* can inhibit *U. maydis* disease progression by direct interaction.	Lee *et al.* (2009)
Olive plants (*Olea europaea*)	*Pseudomonas savastanoi* pv. *savastanoi* (olive knot pathogen) and *Erwinia toletana* (olive knot cooperator).		The bacteria stabilize the community, exchange QS signals, and this cooperation results in disease aggression.	Caballo-Ponce *et al.* (2018)
*Arabidopsis thaliana*	Basidiomycete yeast, *Dioszegia* sp.	*Albugo laibachii*	Construction of an extensive phyllosphere microbial network encompassing bacterial, fungal, and oomycetal communities. Presence of *Dioszegia* sp. is positively correlated with that of *A. laibachii.*	Agler *et al.* (2016)
*Arabidopsis thaliana*	Basidiomycete yeast, *Moesziomyces albugensis*	*Albugo laibachii*	*Moesziomyces albugensis* antagonizes *A. laibachii* on the host leaf surface.	Eitzen *et al.* (2020)
**Innate immunity interaction**				
*Arabidopsis thaliana*	Bacteria	–	The author showed evidence of ethylene signaling (*ein2*) affecting the abundance of *Variovorax*.	Bodenhausen *et al*, (2014)
*Arabidopsis thaliana*	Bacteria	–	Affected diversity of *Firmicutes* sp. and *Proteobacteria* sp. in *min7 fls2 efr cerk1* (*mfec*) and *constitutively activated cell death1* (*cat1*) mutants (involving PTI, MIN7 vesicle trafficking, or cell death pathways).	Chen *et al.* (2020)
*Arabidopsis thaliana*	*Streptomyces* AgN23.	*Alternaria brassicicola*	The bacteria *Streptomyces* induces defense responses, which prevents *Alternaria* infection.	Vergnes *et al.* (2020)
Tomato (*Solanum lycopersicum*, *Solanum pimpinellifolium*)	Bacteria	–	Host resistance shapes leaf microbiota under environmental fluctuations and is time dependent.	Morella *et al.* (2020)
Cucumber *Cucumis sativus* (Suyan 10)	Bacteria and fungi	*Pseudomonas syringae* pv. *Lachrymans*	Plant-specific microbes such as *Sphingomonas*, *Methylobacterium*, *Pseudomonas*, and *Alternaria* are significantly affected by the causal agent of angular leaf-spot of cucumber at different infection stages.	Luo *et al.* (2019)
Pepper (*Capsicum annuum* L.)	*Bacillus thuringiensis*	–	Significant changes of phyllosphere microbiota in *Firmicutes* and *Gammaproteobacteria.*	Zhang *et al.* (2008)
Grapevine (*Vitis vinifera*)	Bacteria	*Botrytis cinerea*, *Phytophthora infestans*	Potential biocontrol agents (*Bacillus*, *Variovorax*, *Pantoea*, *Staphylococcus*, *Herbaspirillum*, *Sphingomonas*) from leaf microbiome acting against phytopathogens.	Bruisson *et al.* (2019)
Wheat (*Triticum aestivum*)	Bacteria and fungi	*Zymoseptoria tritici*	Microbial dynamics upon infection	Kerdraon *et al.* (2019
Tobacco (*Nicotiana* sp.)	Bacteria	*Pseudomonas syringae* pv*. tabaci*	The application of two BCAs changed the bacterial phyllosphere community and decreased bacterial wildfire outbreak.	Qin *et al.* (2019)

Effects of biocontrol agents (BCAs) on crops such as potato against biotrophic (*P. infestans*) and grapevine against necrotrophic (*B. cinerea*) fungi have been studied extensively *in vitro* (Bailly and Weisskopf, 2017; De Vrieze *et al.*, 2018; Bruisson *et al.*, 2019). In contrast, applying *P. syringae* pathovar tomato (*Pst*) to Arabidopsis roots attracted *Bacillus subtilis* and led to IP upon *Pst* infection (Rudrappa *et al.*, 2008; Vannier *et al.*, 2019). The ecological impact of BCAs on the leaf microbiome while controlling disease resistance remains an open research question. Current reports emphasize a linkage between certain bacterial taxa (*Bacillus*, *Pantoea*, *Sphingomonas*, *Pseudomonas*, and *Trichoderma*) affecting microbial diversity (Zhang *et al.*, 2008; Bruisson *et al.*, 2019; Ulrich *et al.*, 2020) and IP induction on leaves (Cawoy *et al.*, 2014; Ritpitakphong *et al.*, 2016; Qin *et al.*, 2019). In particular, highly diverse leaf communities are negatively correlated with pathogen invasion and colonization, and vice versa (Purahong *et al.*, 2018; Qin *et al.*, 2019). Other reports describe difficulties encountered in the application of biocontrol agents such as *B. subtilis*, which did not alter the microbial leaf community under rainy field conditions (Wei *et al.*, 2016). Thus, use of biocontrol agents under natural conditions might be challenging and require further analysis. However, BCAs and IP-inducing compounds can potentially be used to monitor disease control to improve crop yield and production in new biological breeding strategies. There is clearly a need to increase efforts in this research field to explore the effects and underlying mechanisms of abiotic and biotic stress on IP and how they are transmitted to microbial leaf communities.

## Conclusion and outlook

The plant phyllosphere is a highly competitive and challenging habitat for microbes to colonize. Pre-formed barriers such as the hydrophobic cuticle, stomata, or cell wall structures require specific adaptation for the microbes, and persistence of microbes strongly depends on their ability to interact with others. Thus understanding microbiota assembly and persistence in the plant phyllosphere requires investigating ecological factors that shape pre-formed plant structures and therefore directly act on host–microbe and indirectly microbe–microbe interactions. Microbe–microbe interactions in turn not only impact microbial behavior but can impact host fitness by antagonizing plant pathogens. Pathogen invasion generally has a significant negative effect on host fitness caused by tissue damage, nutrient loss to invaders, and reallocation of resources to immune activation. Successful pathogenesis on the other hand is a complex process that requires multiple steps of host colonization and reproduction, and is generally the result of long-term co-evolution (Hall *et al.*, 2017). Considering the enormous gene pool and diversity of all non-pathogenic microbes that are associated with the phyllosphere and other parts of the plant, and considering this pool under constant selection to benefit plant fitness directly or indirectly, we can expect an enormous unexploited pool of beneficial microbes to antagonize pathogenicity processes. In addition, phyllosphere-colonizing microbes are highly adapted to abiotic and biotic fluctuations and are therefore an enormous pool for new adaptive traits.

The downside is, however, that this pool is highly dynamic and probably requires a stable co-existence of different microbial species in one habitat in order to express beneficial traits. Interconnected networks between organisms can be an important element in providing a buffer against perturbations since such links help to recruit microbes to fulfill specific functions in cases where another organism that, for example, provides important antimicrobial compounds or enzymes within the network is lost.

A main goal to develop strategies to protect the phyllosphere from pathogen invasion, such as wheat from rusts, is to identify probiotics that can either stabilize the natural community or become stable on its own. One major effort to develop such probiotics is therefore understanding the multistep process of establishing a niche and defending this niche. Only once we know how to combine traits for stability with our desired traits such as plant protection will we be able to develop products that can replace the majority of our current agrochemicals.
